# Orbital Defect and Emphysema After Nose Blowing: A Case Report and Literature Review

**DOI:** 10.7759/cureus.32958

**Published:** 2022-12-26

**Authors:** Jack J Komro, Parker J Williams, Daniel J Lin

**Affiliations:** 1 Ophthalmology, Ascension St. John Hospital, Detroit, USA; 2 Ophthalmology, Wayne State University, Detroit, USA; 3 Ophthalmology, Central Michigan University College of Medicine, Mount Pleasant, USA

**Keywords:** sneeze, review, orbital fracture, literature review, forceful, emphysema

## Abstract

A 59-year-old man with a history of obstructive sleep apnea presented to the emergency department for acute swelling of the left upper and lower eyelids after nose blowing. The patient denied prior orbital trauma or surgery and examinations were unremarkable for bony step-offs, lacerations, enophthalmos, proptosis, hypoglobus, or extraocular muscle restriction. Imaging confirmed the diagnosis of left anteromedial orbital floor defect with periorbital emphysema. The orbital floor fracture repair was successfully performed with a MEDPOR implant (Stryker, Kalamazoo, Michigan) to seal the persistent orbital floor defect. A review of the literature revealed common predisposing factors, including forceful nose blowing, remote history of trauma, mucosal inflammation, and smoking.

## Introduction

Orbital fractures are becoming more frequent in the United States and are most commonly precipitated by physical trauma [1]. Although rare, orbital wall fractures have been associated with increased intranasal pressure seen in nose blowing and sneezing; however, the predisposing factors leading to this type of morbidity are less clear in the existing literature. Herein, we highlight a novel case of acute orbital defect and emphysema after nose blowing and present a succinct literature review of 17 other cases to better define this rare presentation.

## Case presentation

A 59-year-old white male presented to the emergency department (ED) with acute eyelid swelling of the left eye immediately after sneezing. Pertinent medical history included hypertension, hypercholesterolemia, type 2 diabetes mellitus, obstructive sleep apnea (OSA) with eight years of continuous positive airway pressure (CPAP) use, prior cerebrovascular accident, and gastroesophageal reflux disease. The patient also reported being involved in a mild motor vehicle accident (MVA) approximately 10 years prior without relevant sequelae. The surgical history was unremarkable. Home medications included amlodipine, aspirin, citalopram, pantoprazole, metformin, metoprolol, magnesium oxide, and potassium chloride. Social history was significant for daily alcohol and tobacco pipe use.

The patient reported feeling at his baseline prior to the incident. In the ED, he described having an uneventful morning until he blew his nose. The patient denied ocular trauma, eye pain, pain with extraocular movements, changes in vision, headache, sore throat, numbness, tingling, lightheadedness, or loss of consciousness.

Physical exam was notable for non-erythematous left upper and lower eyelid swelling with crepitus and mild swelling of the left upper maxillary region without pain. The patient’s visual acuity was not tested by the emergency medicine physician because the patient denied any vision changes. Pupils were equal, round, and reactive to light. Intraocular pressure of the affected eye was 17 mmHg. Extraocular movements were full without restriction, proptosis, or enophthalmos. Anterior segment exam and the remainder of the head, eyes, ears, nose, and throat (HEENT) examination was unremarkable, including the absence of scalp or facial lesions or tenderness, patent ear and nasal canals without erythema or edema, no lesions or erythema of the mouth or pharynx, or palpable lymph nodes.

**Figure 1:**
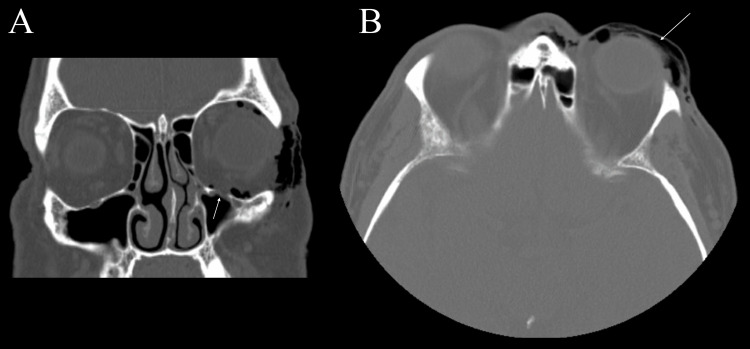
Maxillofacial CT scans without contrast A. Coronal view highlighting a small 5 mm anteromedial orbital floor defect (white arrow) with orbital emphysema and no orbital content herniation or depression fracture. B. Axial view highlighting extensive orbital emphysema (white arrow).

Labs revealed only a mild anion gap metabolic acidosis. A maxillofacial computed tomography (CT) without contrast revealed extensive orbital emphysema on the left and a bony defect involving the anterior medial margin of the left orbital floor without boney fragments (Figure 1). There was no evidence of an orbital floor depression fracture or compromise of the medial, lateral, or superior orbital walls. The paranasal sinuses revealed mild mucosal thickening change involving the left maxillary sinus. The ostiomeatal complexes were patent.

**Figure 2:**
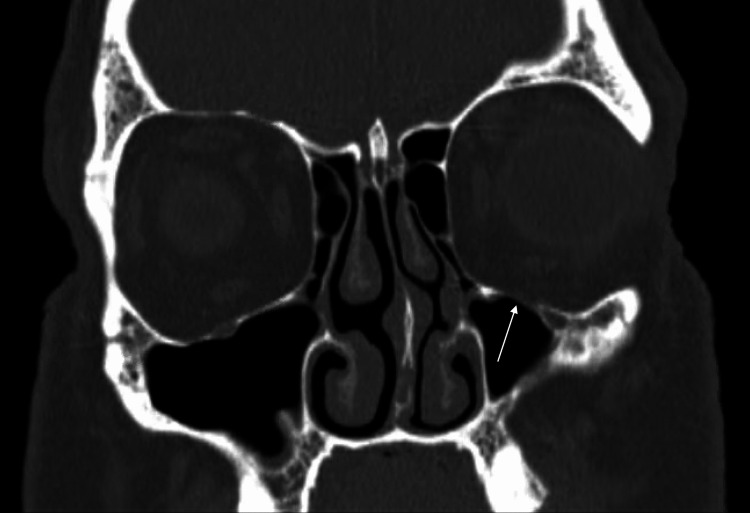
Coronal view CT of the orbits and sella without contrast one month after presentation There is persistence of the anteromedial orbital floor defect (white arrow), with the resolution of orbital emphysema.

The patient was ultimately discharged from the ED on oral amoxicillin-clavulanate and instructed to follow up with an oral and maxillofacial surgeon. Repeat imaging one month later with CT of the orbits and sella without contrast showed resolution of the left-sided orbital emphysema, but persistence of the orbital floor defect along the anterior medial margin (Figure 2). Of note, the paranasal sinuses were clear without mucosal thickening on repeat imaging.

Approximately four months after the initial presentation to the ED, the patient was referred to oculoplastic surgery for repair of the orbital floor defect. Visual acuity at the initial presentation to our office was 20/25 in both eyes and intraocular pressures were normal. The examination mirrored the prior exams. Orbital floor fracture repair of the left eye was successfully achieved using a MEDPOR implant (Stryker, Kalamazoo, Michigan) to seal the defect; however, the clinical course was complicated by retrobulbar hemorrhage secondary to premature blood thinner use on the third postoperative day that required lateral canthotomy and inferior cantholysis. At the six-month follow-up, the implant remained in a good position with a resolution of symptoms and return to baseline visual acuity of 20/25 in both eyes.

## Discussion

Orbital fractures from all causes most commonly occur in the medial wall, followed by the floor and inferomedial wall [2]. Orbital floor fractures, as in our case, have been well-documented related to physical trauma [1,2]. In a study by Iftikhar et al. (2021) spanning from 2006 to 2017, the two most common causes of orbital floor fractures presenting to the ED were assault (43%) and falls (26%), followed by objects and MVAs [1]. Non-physical causes of orbital floor defects, such as nose blowing and barotrauma, account for a minority of cases.

A literature review was conducted through PubMed and reference lists from January 1, 1996, to January 1, 2022, for reports of non-traumatic orbital wall fractures associated with nose blowing. Case reports were excluded if they were related to acute trauma, recent surgery within five years, or lack of a fracture. A total of 17 additional cases met the inclusion and exclusion criteria to review, for a total of 18 cases (Table 1) [3-19]. All of the cases involved fractures of either the medial wall [3,5,7-12] or the floor [4,6,13-19], which, anatomically, are the thinnest locations of the orbital walls. The average age on presentation was 45.2 years old (range: 30 to 76 years). The majority of these patients were female (11/18, 61.1%) [2,5,6,8-10,12,13,15-17]. All patients (18/18, 100%) presented with swelling of the eyelids or periorbital region after a nose-blowing episode [3-19]. Nine cases (9/17, 52.9%) specifically mentioned “strong,” “vigorous,” or “forceful” nose blowing prior to symptom onset [4-6,8,10,12,14,16,18]. On CT imaging, just over half of the fractures involved the orbital floor (10/18, 55.6%) [4,6,13-19]. Altogether, nine patients (9/18, 50.0%) had CT evidence of some degree of herniation of orbital contents [3-6,8-10,13,15] and 17 had documented orbital emphysema (17/18, 94.4%) [3-16,18,19].

**Table 1:** A literature review of nose-blowing-associated orbital fractures VA = visual acuity; IOP = intraocular pressure; EOM = extraocular movements; CPAP = continuous positive airway pressure; M = male; F = female; Y = yes; N = no.

Publication	Age (years)	Sex (M/F)	Fracture location	Etiology	Eyelid swelling	Pain	Decreased VA	Increased IOP	Limited EOM	Presentation	History	Computed tomography findings
Kim et al. (2021) [3]	40	F	Medial wall	Nose blowing	Y	Y	N	N	N	Sudden painful swelling of the eyelid	Prior open reduction and internal fixation for left zygomatic fracture with insertion of mesh plate (for an impure orbital floor blowout fracture six years prior)	Medial wall blowout fracture measuring 11 x 8 mm with subcutaneous emphysema (extraconal fat herniated through the fracture site)
Sarbajna et al. (2020) [7]	76	M	Medial wall	Nose blowing	Y	N	Y	Y	Y	Eyelid swelling, decreased vision, increased IOP, limited EOM	Unspecified	Medial orbital wall fracture with severe emphysema
Ariyoshi et al. (2019) [8]	59	F	Medial wall	Nose blowing	Y	N	N	N	N	Sudden, painless left periorbital swelling	Chronic rhinitis	Orbital subcutaneous and subconjunctival emphysema, fracture of the medial orbital wall of the left eye, focal herniation of extraconal fat into ethmoid air cells
Myers et al. (2018) [9]	36	F	Medial wall	Nose blowing	Y	Y	N	N	N	Sudden onset bleeding from the left nostril two hours after blowing nose, left eye swelling, stabbing pain in the left side of the head and back (radiating to left arm)	Fit and well with no previous medical history, smoking one pack per day cigarettes	Lamina papyracea fracture with focal herniation of extraconal fat into ethmoid air cells and slight tenting of medial rectus muscle toward the defect, extraconal orbital emphysema was present
Mohebbi et al. (2017) [13]	38	F	Floor	Nose blowing	Y	N	N	N	N	Periorbital swelling	Seasonal allergies	Subcutaneous emphysema, left orbital floor blowout fracture, herniation of orbital fat/inferior rectus/inferior oblique into the maxillary sinus
Hu et al. (2017) [14]	33	M	Floor	Nose blowing	Y	N	N	N	N	Periorbital swelling, erythema	Allergic rhinitis	Orbital emphysema of left eye, fracture of the left orbital floor into the maxillary sinus, prolapse of orbital soft tissue into the left maxillary sinus
Sandhu et al. (2016) [15]	40	F	Floor	Nose blowing	Y	Y	Y	N	N	Left eye pain, periorbital edema, blurred vision, 10/10 headache	Migraine headache, gastritis	Comminuted left orbital floor fracture with herniation of orbital fat, fracture fragments, and blood within the left maxillary sinus, and preseptal and extraconal orbital emphysema
Jawaid (2015) [16]	32	F	Floor	Nose blowing	Y	N	N	N	N	Eyelid swelling, eyelid emphysema	Nothing significant, per the authors	Fracture of the right orbital floor into the maxillary sinus and nasal bone, periorbital emphysema
Hwang et al. (2014) [10]	35	F	Medial wall	Nose blowing	Y	N	N	N	N	Swelling of the left orbital region	Unspecified	Blowout fracture of medial wall of left orbit with some herniation of orbital soft tissue into the ethmoidal sinus and subcutaneous emphysema in left eyelids and cheek
Watanabe et al. (2012) [4]	30	M	Floor	Nose blowing	Y	N	N	N	Y	Eyelid edema, diplopia	Unspecified	Blowout fracture of inferior orbital wall, orbital and subcutaneous emphysema, herniation of orbital soft tissue into the maxillary sinus
Halpenny et al. (2012) [17]	49	F	Floor	Nose blowing	Y	Y	N	N	N	Facial swelling, facial tenderness, periorbital hematoma	No history of acute facial trauma, +history of trauma to the left side of the face 10 years prior (but no facial bone fractures were documented at that time), recent frontal headache for several days prior to fracture at presentation (treated as sinusitis)	Fracture of the left orbital floor and lateral wall of the left maxillary sinus
Rahmel et al. (2010) [18]	40	M	Floor	Nose blowing	Y	Y	N	N	N	Periorbital swelling, facial pain, hypoesthesia of the cheek	Eczema, hyperlipidemia, chronic seasonal sinusitis	Comminuted blowout fracture of left orbital floor, extensive subcutaneous emphysema
Rosh et al. (2008) [11]	58	M	Medial wall	Nose blowing	Y	N	N	N	N	Eye swelling	Nothing significant per authors, Cataract surgery of both eyes 10 years prior	Defect in the left lamina papyracea, significant left orbital emphysema, subcutaneous air within soft tissues of the face, extending down to the level of the maxilla with mild sinus mucosal disease
Garcia de Marcos et al. (2008) [19]	35	M	Floor	Nose blowing	Y	N	N	N	N	Orbital emphysema	Unspecified	Left orbital floor fracture, polyploid lesion in the ipsilateral maxillary sinus, orbital emphysema
Garcia-Medina et al. (2006) [12]	51	F	Medial wall	Nose blowing	Y	Y	N	N	N	Painful periorbital swelling	None	Left proptosis, extensive confluent orbitopalpebral emphysema, blowout fracture of left orbital medial wall
Suzuki et al. (2001) [5]	32	F	Medial wall	Nose blowing	Y	N	N	N	N	Orbital swelling	Unspecified	Blowout fracture of the medial orbital wall with orbital emphysema and herniation of orbital soft tissue
Oluwole et al. (1996) [6]	70	F	Floor	Nose blowing	Y	Y	N	N	N	Pain and swelling of the left eye	Hip replacement for osteoarthritis	Orbital emphysema, early orbital cellulitis, entrapment of left inferior rectus muscle, fracture of the left orbital floor with herniation of orbital soft tissue into the maxillary antrum
Our case	59	M	Floor	Nose blowing	Y	N	N	N	N	Acute painless periorbital swelling	Tobacco pipe use, CPAP use, deviated septum	Extensive orbital emphysema on the left and a bony defect involving the anterior medial margin of the left orbital floor without boney fragments
18 cases	45.2	11 females, 7 males	10 medial wall, 8 orbital floor	18 nose blowing	18 yes	11 no, 7 yes	16 no, 2 yes	17 no, 1 yes	16 no, 2 yes			

Regarding past medical history, five patients (5/18, 27.8%) had no history of facial trauma, facial or nasal surgery, sinusitis, or illness [11,13-16]. Prior trauma was documented in two cases (2/18, 11.1%), one of which had been treated with open reduction and internal fixation for an orbital floor blowout fracture six years prior [3], while the other had isolated left-sided facial trauma 10 years prior without fractures [17]. Rhinitis, sinusitis, or seasonal allergies were documented in five patients (5/18, 27.8%) [8,13,14,18]. Three patients (3/18, 16.7%) smoked tobacco regularly [9,19]. Concurrent illness or other upper respiratory infections were noted in four cases (4/18, 22.2%) [6,12,17,19].

Mechanisms for orbital wall fracture after nose blowing were postulated in four reports, which primarily implicate prior facial surgery, boney changes of advanced age, and intensity of nose blowing. Kim et al. (2021) proposed that the patient’s medial wall fracture after nose blowing was secondary to postoperative changes in the aerodynamics or shock-absorbing capacity of the paranasal sinuses after having an orbital floor fracture repair six years prior [3]. Two cases were in agreement that high intranasal force from vigorous nose blowing could cause an orbital wall fracture consistent with the hydraulic theory [4,5]. Finally, Oluwole and White (1996) in the first case report describing an orbital fracture after nose blowing suggested that the fracture seen in the 70-year-old patient was precipitated by a combination of vigorous nose blowing and natural thinning of the bony wall with age [6].

Based on the known literature, potential predisposing factors in our patient’s case include forceful nose blowing, sinusitis, smoking, and a remote history of an MVA; however, it may be possible that CPAP contributed to this presentation. CPAP is known to create dryness and inflammation of the nasal mucosa through continuous airflow. Air travels into the maxillary sinus via the nasal cavity, under the middle concha, and into the middle meatus where the maxillary sinus ostium is located in the hiatus semilunaris. Of note, the primary maxillary ostium is typically located at the junction of the medial maxillary wall and orbital floor, halfway between the anterior and posterior maxillary walls [20]. It is obvious how chronic airflow from CPAP through this pathway can create inflammation and gradual thinning of the maxillary sinus roof and orbital floor. Furthermore, based on the anatomy described above, the defect would most likely occur along the medial orbital floor, because it is the initial contact site when air enters the maxillary sinus through the maxillary ostium. The patient presented in this case report had an anteromedial orbital floor defect, which aligns with this potential mechanism. There is likely slight anatomic variation in the anteroposterior location of the maxillary sinus ostium, explaining why the patient’s defect was located more anteriorly.

## Conclusions

Nose blowing is an established cause of orbital fracture and emphysema. Predisposing factors have not been fully elucidated, but after a review of the literature, common predisposing factors include forceful nose blowing, remote history of trauma, mucosal inflammation, and smoking. It is thought that CPAP contributed to mucosal irritation and orbital floor thinning in our patient’s case. No meaningful associations were observed relating to the age or sex of patients. All orbital fractures affected either the nasal wall or the orbital floor, and nearly all cases presented with periorbital emphysema. Our case with a persistent orbital floor defect was successfully repaired using an orbital implant.
